# Comparative postoperative outcomes of GGN-dominant vs single lesion lung adenocarcinomas

**DOI:** 10.1186/s13019-020-01196-x

**Published:** 2020-06-22

**Authors:** Takamasa Hotta, Yukari Tsubata, Akari Tanino, Mika Nakao, Yoshihiro Amano, Megumi Hamaguchi, Shunichi Hamaguchi, Koji Kishimoto, Takeshi Isobe

**Affiliations:** 1 Division of Medical Oncology & Respiratory Medicine, Department of Internal Medicine, Shimane University, 89-1 Enya-cho, Izumo, Shimane 693-8501 Japan; 2 Division of Thoracic Surgery, Shimane University, 89-1 Enya-cho, Izumo, Shimane 693-8501 Japan

**Keywords:** Multiple primary lung cancers, Adenocarcinoma, Ground glass opacity, Ground glass nodule, Surgical treatment

## Abstract

**Background:**

Multiple synchronous ground glass nodules (GGNs) are known to be malignant, however, they tend to progress slowly. Multiple synchronous lesions in the same patient which show different characteristics must be treated individually.

**Methods:**

This was a retrospective review of 34 lung adenocarcinoma patients with multiple synchronous GGNs in an Asian population. One hundred twenty-seven single lung adenocarcinoma patients were included for comparison purposes. The follow-up period was 5 years for all patients.

**Results:**

The 5-year overall survival (OS) patients with multiple lesions did not differ from that of the patients with single lesions to a statistically significant extent (Single: 81.8% vs. Multiple: 88.2%, *P* = 0.3602). Dominant tumors (DTs) with a ground glass component and consolidation were divided into three categories based on the consolidation-to-tumor ratio on radiological imaging. No significant differences were observed among the three DT categories. Twenty-four patients had unresected GGNs, while a progression of the unresected GGN occurred in 10 of these cases. The OS and disease-free survival (DFS) curves of patients with and without GGN progression did not differ to a statistically significant extent (OS: 80% vs. 92.9%, *P* = 0.3870; DFS: 80% vs. 100%, *P* = 0.0977).

**Conclusions:**

The outcomes were best predicted by the stage of the DT. After surgery patients require a careful follow-up because unresected GGNs may show progression. At the same time, the increase in residual lesions and the appearance of new GGNs were not related to OS. The management of such patients should be determined according to the DT with the worst prognosis.

## Introduction

Low-dose computed tomography (CT) screening has led to a relative reduction in mortality from lung cancer [1], and an increase in the incidental diagnosis of small pulmonary ground glass nodules (GGNs) [2–5]. New guidance on the management of GGN is required and has been proposed [6, 7]. However, no standard algorithms have been established for multiple GGNs detected by screening. Thus, there is a lack of clinical evidence on their natural history, diagnosis and treatment.

A number of studies have suggested that multiple GGNs have independent characteristics [8, 9]. Next-generation sequencing has shown that some multiple synchronous lesions show different mutation profiles in the same patient, while others share identical gene mutations [10]. These results suggest that the dominant tumor (DT) and synchronous GGNs are genetically independent tumors. Evidence is emerging that—given the independent characteristics of each of the multiple GGNs in a patient—the management of multiple GGNs should be determined based on the DT that carries the worst prognosis [11, 12]. Follow-up of the remaining lesions after the surgical treatment of the DT has been reported [13, 14]. However, there no reports have compared the outcomes of patients with multiple GGNs to those of patients with single lesions. Furthermore, in cases of synchronous GGNs, the postoperative outcomes were compared according to the progression of DT in order to investigate whether priority should be given to the DT when deciding the treatment strategy.

## Patients and methods

We retrospectively studied patients who were referred for surgery at Shimane University Hospital, from January 2009 to December 2013. Two hundred seven patients met the following criteria: [1] adenocarcinoma, and [2] pN0. Pre-operative CT scans were reviewed to identify synchronous GGNs. Thirty-nine patients had one or more lesions other than the DT. The DT was defined as the lung lesion of the largest diameter or the lesion that showed the most radiological invasiveness (margin of the nodule, pleural indentation, presence of a solid component).

**Fig. 1:**
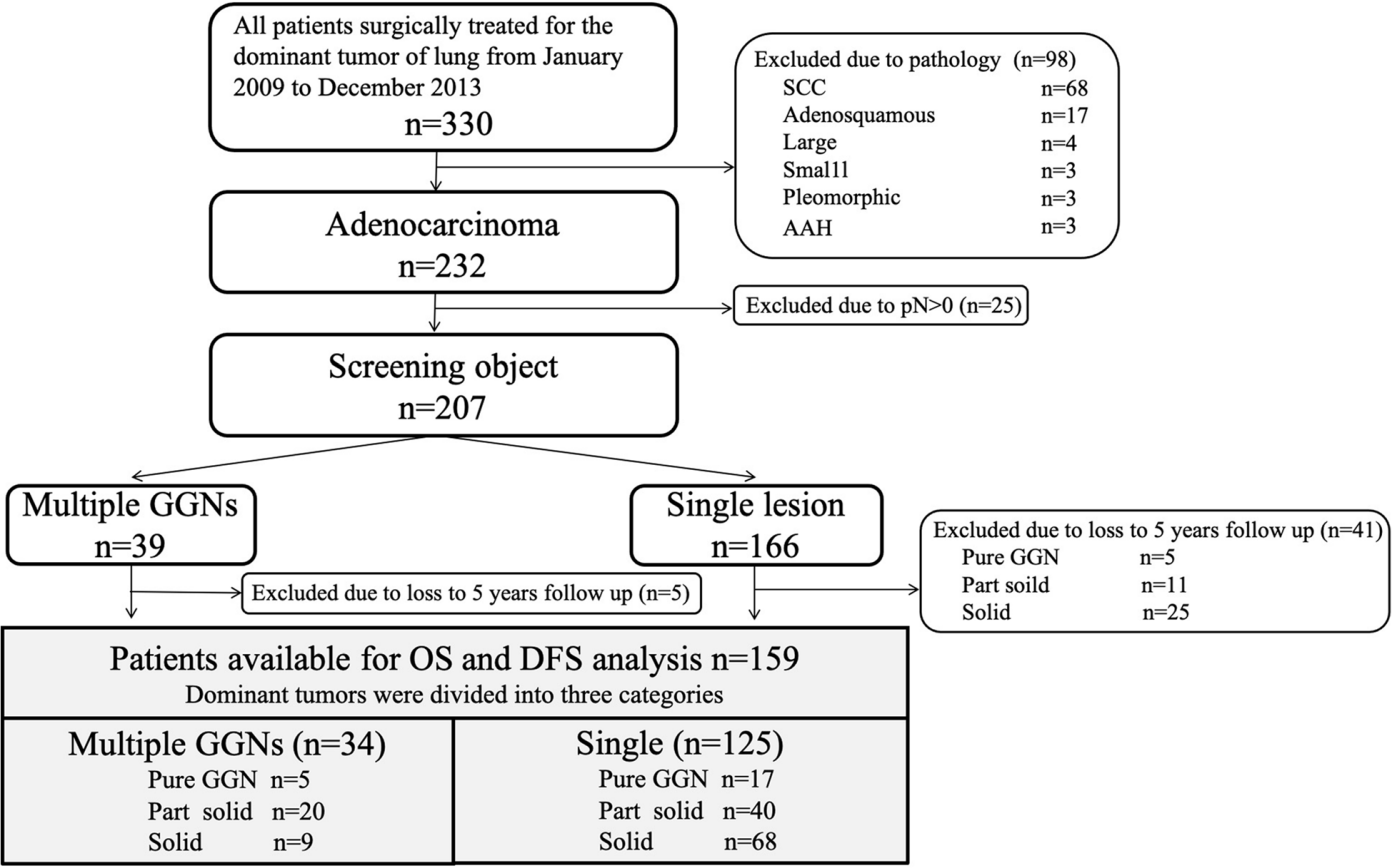
A flow diagram of the present study. GGNs; ground glass nodules, SCC; squamous cell carcinoma, AAH; atypical adenomatous hyperplasia, OS; overall survival, DFS; Disease-free survival

DTs with a ground glass component and consolidation were divided into three categories based on the consolidation-to-tumor (C/T) ratio on radiological imaging: pure GGN (C/T ratio = 0), part solid (C/T ratio > 0 to < 1) and solid tumor (C/T ratio = 1). The following cases were excluded from analysis: [1] cases involving recurrent lung cancer or in which the outcome was unknown, [2] stable lung cancer cases for which CT data had not been obtained for 5 years after surgery (Fig. 1). Radiological interpretation and date of recurrence were taken from the medical records as judged by two radiologists.

Lung cancers were staged in accordance with the seventh edition of the TNM Classification for Lung and Pleural Tumors. Echocardiography, spirometry, and lower limb echo in patients with a high d-dimer level were performed as preoperative tests. The preoperative CT-guided hookwire localization for pulmonary nodules, particularly for GGNs, was used. The operations typically removed the DT along with any accessible ipsilateral GGNs. Lobectomy with complete systematic lymph node dissection was the standard surgical treatment. Limited resection was applied for patients with severe complications and for the elderly, especially those presenting with pure GGNs. Lung adenocarcinomas were described pathologically as adenocarcinoma in situ, minimally invasive adenocarcinoma, invasive adenocarcinoma or variants of invasive adenocarcinoma. Slides were reviewed by a pathologist to confirm the presence of a lepidic component.

All patients were regularly evaluated by CT every 3 months for the first 2 years after surgery and every 6 months thereafter. Tegafur-uracil was selected as adjuvant treatment for T1b patients. Cisplatin-based adjuvant chemotherapy was selected for patients with stage II disease. Non-dominant GGNs that were followed were generally treated by either surgical resection or stereotactic radiotherapy (SRT), when they grew size with any solid component.

The patient and tumor characteristics were analyzed to identify factors associated with overall survival (OS), disease-free survival (DFS), and progression of GGN. OS was calculated from the date of surgery until either death from any cause or the date of last follow-up, with a minimum of 5 years of study inclusion. DFS was defined as survival without extrapulmonary metastasis, locoregional or distant recurrence, or GGN progression requiring intervention. GGN progression was defined as growth of a GGN, development of a new solid component in a pure GGN, or enlargement of a solid component in a part-solid GGN with stable total diameter. Considering the error due to CT slice thickness, growth was defined as an increase of ≥5 mm.

### Statistical analyses

Statistical analyses were performed using the GraphPad Prism 7 software program (GraphPad Software, La Jolla, CA, USA). Qualitative variables were reported the frequency and percentage, while quantitative variables were reported as the mean and standard deviation. Comparisons between two groups were performed using the unpaired *t*-test for normally distributed data. Categorical variables were compared using Fisher exact test. OS and DFS were calculated from the date of surgery and estimated using a Kaplan-Meier analysis. *P* values of < 0.05 were considered to indicate statistical significance.

## Results

### Patient characteristics

DFS and OS analyses were performed in 159 patients (multiple GGNs [*n* = 34] vs Single lesion [*n* = 125]). Dominant tumors were divided into three categories (Pure GGN, Part solid and Solid). Patients with multiple GGNs were divided into pure [*n* = 5], partly solid [*n* = 20] and solid [*n* = 9). Patients with a single lesion were divided into pure [*n* = 17], partly solid [*n* = 40] and solid [*n* = 68) (Fig. 1). The patient and surgical characteristics are summarized in Table 1. There were 159 patients (multiple GGN [*n* = 34], pure GGN [n = 17], part solid [n = 40] and solid [n = 68]). Five years of follow-up was completed in all cases. All patients were Asians and underwent video-assisted thoracoscopic resections. Combined resection of multiple lesions was performed for 23% of the patients in the multiple GGN group. Twenty percent of the patients underwent wedge resection alone. Fatal complications occurred in 2 patients in the solid group due to pulmonary embolism and a pulmonary artery rupture. Histologically, no invasive cancer was found in the Pure GGN group. Chemotherapy was performed according to the DT tumor size (pStage). There were three patients with pStage IIa disease in the multiple GGN group who did not receive chemotherapy.

**Table 1:** Patient characteristics

	Multiple (*n* = 34)	Single		
		Pure GGN (*n* = 17)	Part Solid (*n* = 40)	Solid (*n* = 68)
Age, (years)	73.7 ± 7.9	65.0 ± 11.2	71.3 ± 13.0	71.6 ± 9.4
Gender, (male/female), *n*	18/16	8/9	22/18	31/37
Previous cancer history, (Yes), *n*	3 (9)	4 (24)	11 (28)	13 (19)
Surgical procedure, *n*				
Operation				
Open Thoractomy	0	0	0	0
Video-assisted thoracoscopy	34 (100)	17 (100)	40 (100)	68 (100)
Resection type				
Wedge resection	7 (20)	10 (59)	5 (12)	4 (6)
Segmentectomy	0	0	3 (8)	1 (1)
Lobectomy	19 (37)	7 (41)	32 (80)	62 (92)
Combination	8 (23)	0	0	1 (1)
Fatal complications	0	0	0	2 (3)
Histology, *n*				
Adenocarcinoma in situ	15 (44)	15 (88)	9 (22)	1 (1)
Minimally-invasive adenocarcinoma	1 (3)	2 (12)	3 (8)	0
Invasive adenocarcinoma	17 (50)	0	26 (65)	59 (87)
Variants of invasive adenocarcinoma	1 (3)	0	2 (5)	8 (12)
Chemotherapy, *n*				
None	27 (79)	17 (100)	31 (78)	32 (47)
UFT	6 (18)	0	9 (22)	31 (46)
Cisplatin based chemotherapy	1 (3)	0	0	5 (7)
Dominant Tumor Size (mm)	23.7 ± 13.9	10.9 ± 4.6	19.4 ± 7.9	28.8 ± 20.1
pStage Ia, *n*	24 (70)	17 (100)	35 (87)	40 (59)
Stage Ib, *n*	6 (18)	0	4 (10)	23 (34)
Stage IIa, *n*	4 (12)	0	1 (3)	4 (6)
pStage IIb, *n*	0	0	0	1 (1)

Values are means ± standard deviations or number, number (percentage). *GGN* ground glass nodule, *UFT* tegafur-uracil

### Postoperative outcomes

Table 2 lists the results of postoperative surveillance. There were 17 cases of postoperative recurrence (multiple [*n* = 2], part solid [*n* = 3], and solid [*n* = 12]). Eleven of them were positive for epidermal growth factor receptor (EGFR) mutations. The OS decreased as the C/T ratio increased (pure GGN, 94.1%; part solid, 90%; solid, 75%; Table 2).

**Table 2:** The results of postoperative surveillance

	Multiple			Single		
	Pure GGN (*n* = 5)	Part Solid (*n* = 20)	Solid (*n* = 9)	Pure GGN (*n* = 17)	Part Solid (*n* = 40)	Solid (*n* = 68)
Recurrent patients, *n*	0	1 (5)	1 (11)	0	3 (8)	12 (18)
Mutation sutatus, *n*						
EGFR (+)	0	1 (100)	1 (100)	0	1 (33)	7 (62)
EGFR (−)	0	0	0	0	1 (33)	2 (15)
Unknown	0	0	0	0	1 (33)	3 (23)
Histology, *n*						
Adenocarcinoma in situ	0	0	0		1 (33)	0
Invasive adenocarcinoma	0	1 (100)	1 (100)		2 (67)	12 (100)
5-year overall survival, %	100	90	77.8	94.1	90	75
5-year disease-free survival, %	100	95	87.5	100	92.2	80.2

Values are the number (percentage) or percentage

*GGN* ground glass nodule, *EGFR* epidermal growth factor receptor

**Fig. 2:**
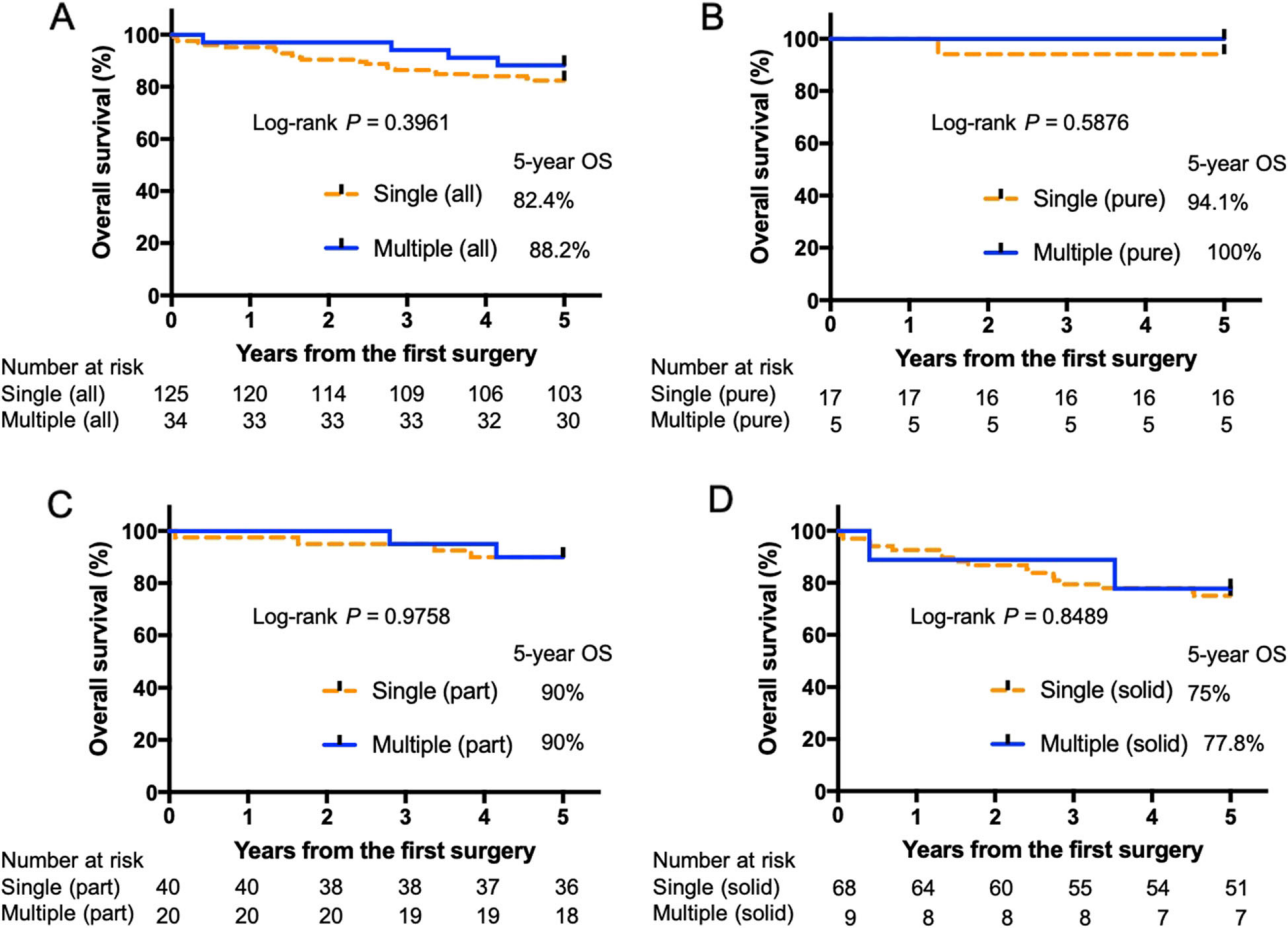
The Kaplan-Meier analysis of the survival of patients with multiple and single lesions. **a** Single: 81.8% vs. Multiple: 88.2%. When classified for each DT and compared (**b**) pure GGN, 94.1% vs. multiple GGN (pure), 100%; **c** part solid, 90% vs. multiple GGN (part), 90%; **d** Solid, 74.3% vs. multiple GGN (solid), 77.8%. GGNs; ground glass nodules, OS: overall survival

**Fig. 3:**
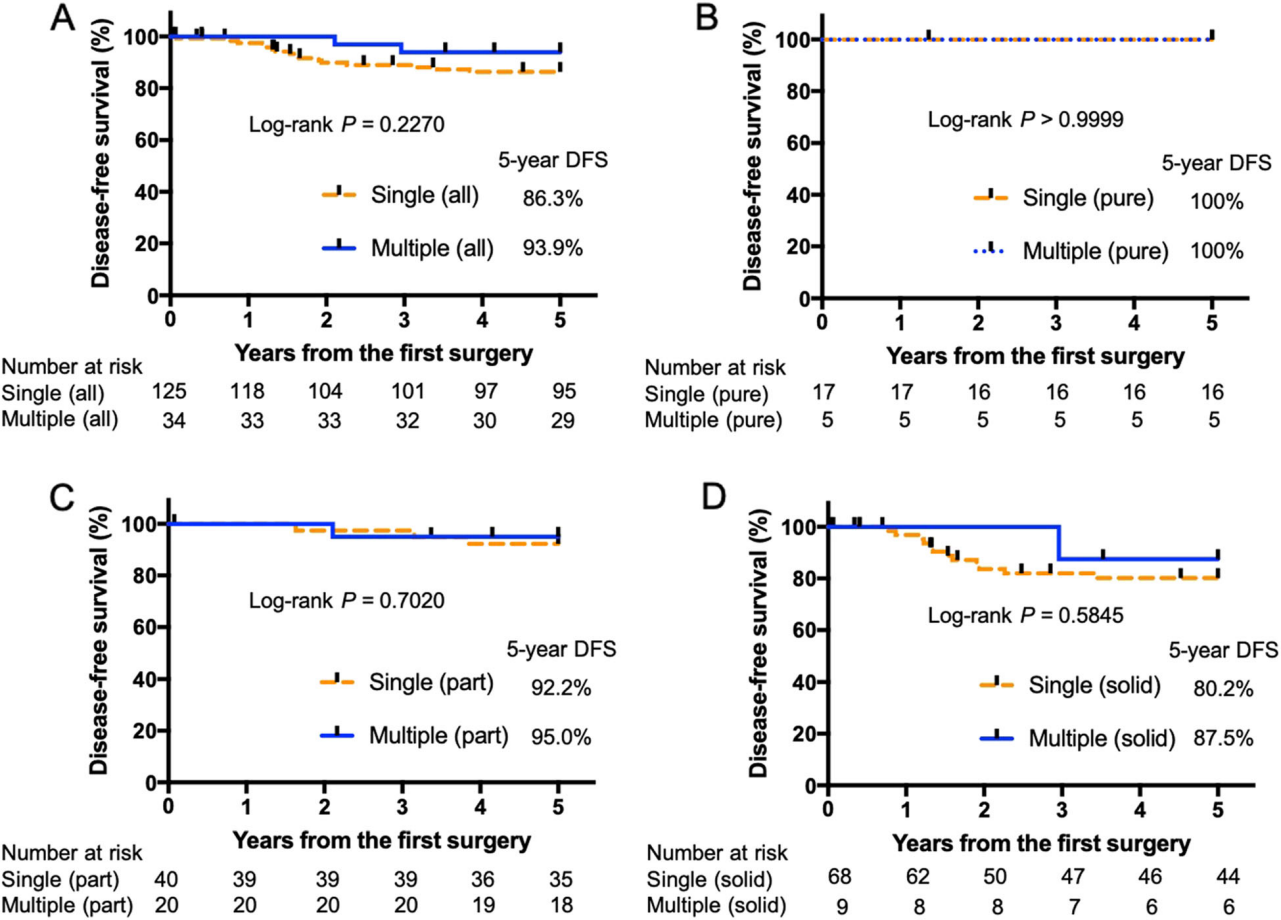
Disease-free survival curves of patients with multiple and single lesions. **a**) single vs. multiple, **b** pure GGN vs. multiple GGN (pure), **c** part solid vs. multiple GGN (part), **d** solid vs. multiple GGN (solid). GGNs; ground glass nodules, DFS; disease free survival

Kaplan-Meier analyses (Fig. 2a) were performed to compare the patients with multiple lesions to those with single lesions; their survival did not differ to a statistically significant extent (single, 82.4% vs. multiple, 88.2%; *P* = 0.3602). There when the outcomes were compared according to the DT classifications (Pure GGN, 94.1% vs. multiple GGN [pure], 100%; part solid, 90% vs. multiple GGN [part], 90%; solid, 75% vs. multiple GGN [solid]: 77.8%; Fig. 2b–d). The DFS was similar to the OS (Fig. 3).

### Characteristics of the DT and non-dominant GGNs in the multiple GGN group

**Fig. 4:**
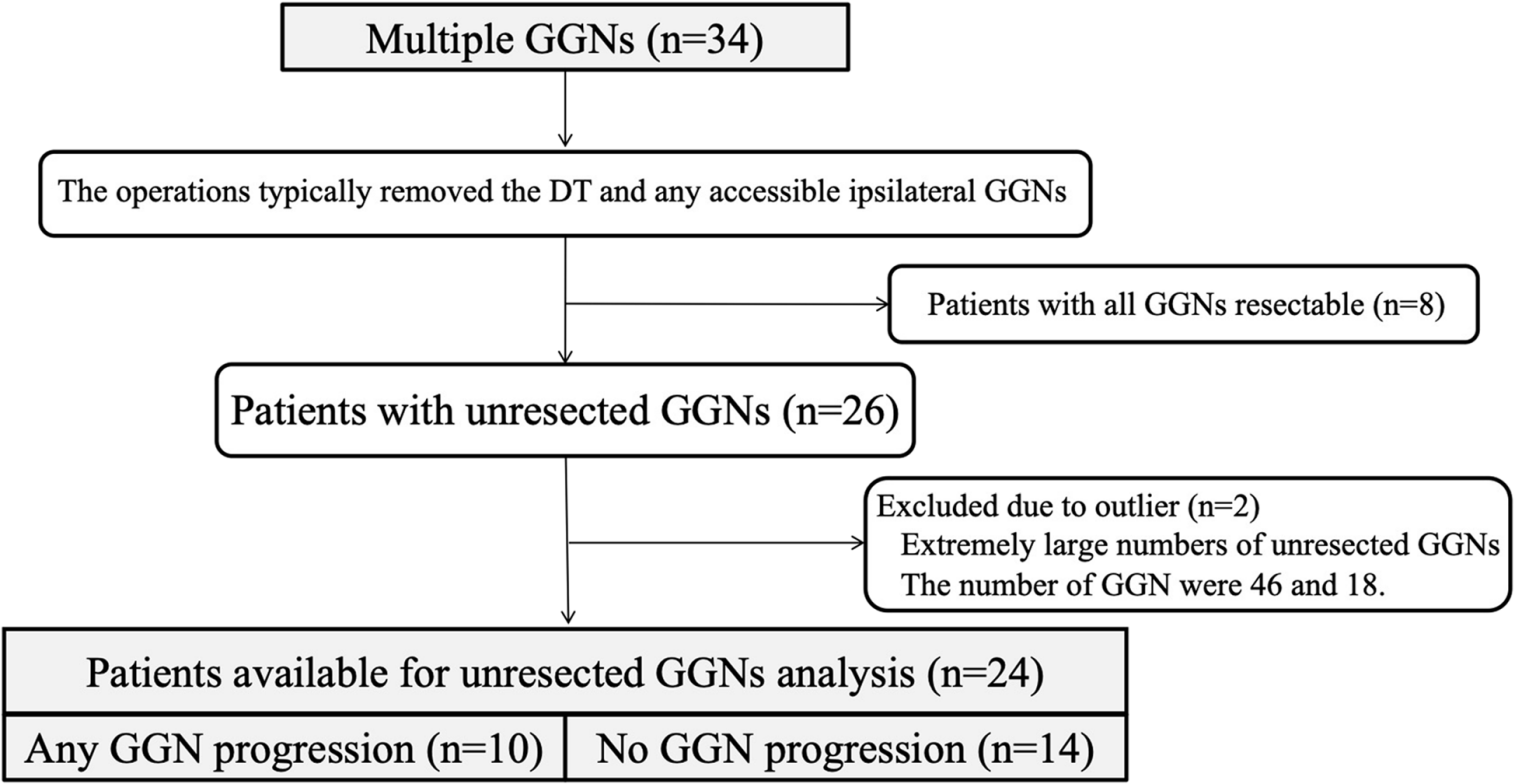
A flow diagram of the patients with unresected GGN. GGNs; ground glass nodules

Characteristics of the DT and the non-dominant GGN are summarized in Table 3. As for non-dominant GGN, there were 2 cases with very large numbers of nodules (46 and 18) in the part solid group; these cases were excluded as outliers. Radiographically, the mean diameters of the DTs were as follows: Pure GGN, 8.6 ± 4.6 mm; part solid, 21.7 ± 11.6 mm; and solid, 36.8 ± 11.2 mm. The mean diameters of the largest non-dominant GGN at presentation were as follows: pure GGN, 8.1 ± 1.8 mm; part solid, 8.2 ± 5.2 mm; and solid, 17.5 ± 11.7 mm. The numbers of with non-dominant GGNs were as follows: pure GGN (range, 1–2), *n* = 8; part solid (range, 1–7), *n* = 38; and solid (range, 1–5), *n* = 17. Four pure GGNs (50%), 19 part solid GGNs (50%) and 9 solid GGNs (53%) were identified in the contralateral lung to the DT. Some of the lesions in the lung lobe that differed from the DT could not be resected. Twenty-six patients had unresected GGNs. Two patients were excluded due to an extremely large numbers of unresected GGNs (*n* = 46 and 18). Finally, 24 patients were included in the unresected GGNs analysis. In 10 of these patients, disease progression was observed in the unresected GGNs (Fig. 4). In 2 cases, additional surgery or SRT was performed to treat the unresected GGN. Most unresected GGNs were pure GGN and none had a C/T ratio of > 0.5.

**Table 3:** Characteristics of patients with multiple GGNs

	Multiple (N = 34)		
	Pure GGN	Part Solid	Solid
Patients, *n*	5	20	9
Dominant Tumor Size (mm)	8.6 ± 4.6	21.7 ± 11.6	36.8 ± 11.2
Nondominant GGN			
Largest GGN size (mm)	8.1 ± 1.8	8.2 ± 5.2	17.5 ± 11.7
Number, *n* (range per patient)	8 (1–2)	38 (1–7)	17 (1–5)
Location per GGN			
Ipsilateral lung			
Same lobe, *n*	2 (25)	7 (18)	6 (35)
Different lobe, *n*	2 (25)	12 (32)	2 (12)
Contralateral lung, *n*	4 (50)	19 (50)	9 (53)
Unresected GGN, *n*	5	22	11
Patients, *n*	3	14	7
Patients with unresected GGN that grew, *n*	0	7	3
Patients with intervention for unresected GGN, *n*	0	2	0
Stereotactic radiotherapy, *n*	0	1	0
Surgical resection, *n*	0	1	0
C/T ratio			
= 0, *n*	5 (100)	19 (86)	11 (100)
0 < C/T ratio < 0.5, *n*	0	3 (14)	0

Values are the mean ± standard deviation, number, number (percentage) or number (range per patient)

*GGNs* ground glass nodules

### Characteristics of the patients with GGN progression

The characteristics of the patients with or without progression of GGN are summarized in Table 4. The mean size of the unresected GGNs in the any GGN progression group was predominantly larger than that in the other group (mean size, 10.3 ± 5.2 mm vs. 6.5 ± 4.3 mm, respectively *P* = 0.0004). The OS curve of the cases with and without GGN progression did not differ to a statistically significant extent (80% vs. 92.9%, *P* = 0.3870).

**Table 4:** Characteristics of patients with GGN progression

	Unresected GGN (+)		
	Progression	No progression	*P*=
	*N* = 10	*N* = 14	
Age, (years)	72.9 ± 8.1	72.9 ± 9.1	0.9656
Gender, (male/female), *n*	5/5	5/9	0.6785
Dominant Tumor Size (mm)	28.4 ± 12.7	20.9 ± 14.5	0.1580
Smoking status (Pack-year)	19.4 ± 21.9	8.3 ± 13.7	0.3280
Dominant Tumor Histology, *n*			0.2138
Adenocarcinoma in situ	3 (30)	9 (64)	
Invasive adenocarcinoma	7 (70)	5 (36)	
Unresected GGN, *n*	20	18	
Mean GGN size (mm)	10.3 ± 5,2	6.5 ± 4.3	0.0004
Largest GGN size (mm)	12.4 ± 6.4	7.2 ± 4.6	0.0681
Survival			
5-year overall survival, %	80	92.9	0.387

Values are the mean ± standard deviation, number, number (percentage) or percentage

*GGN* ground glass nodule

**Fig. 5:**
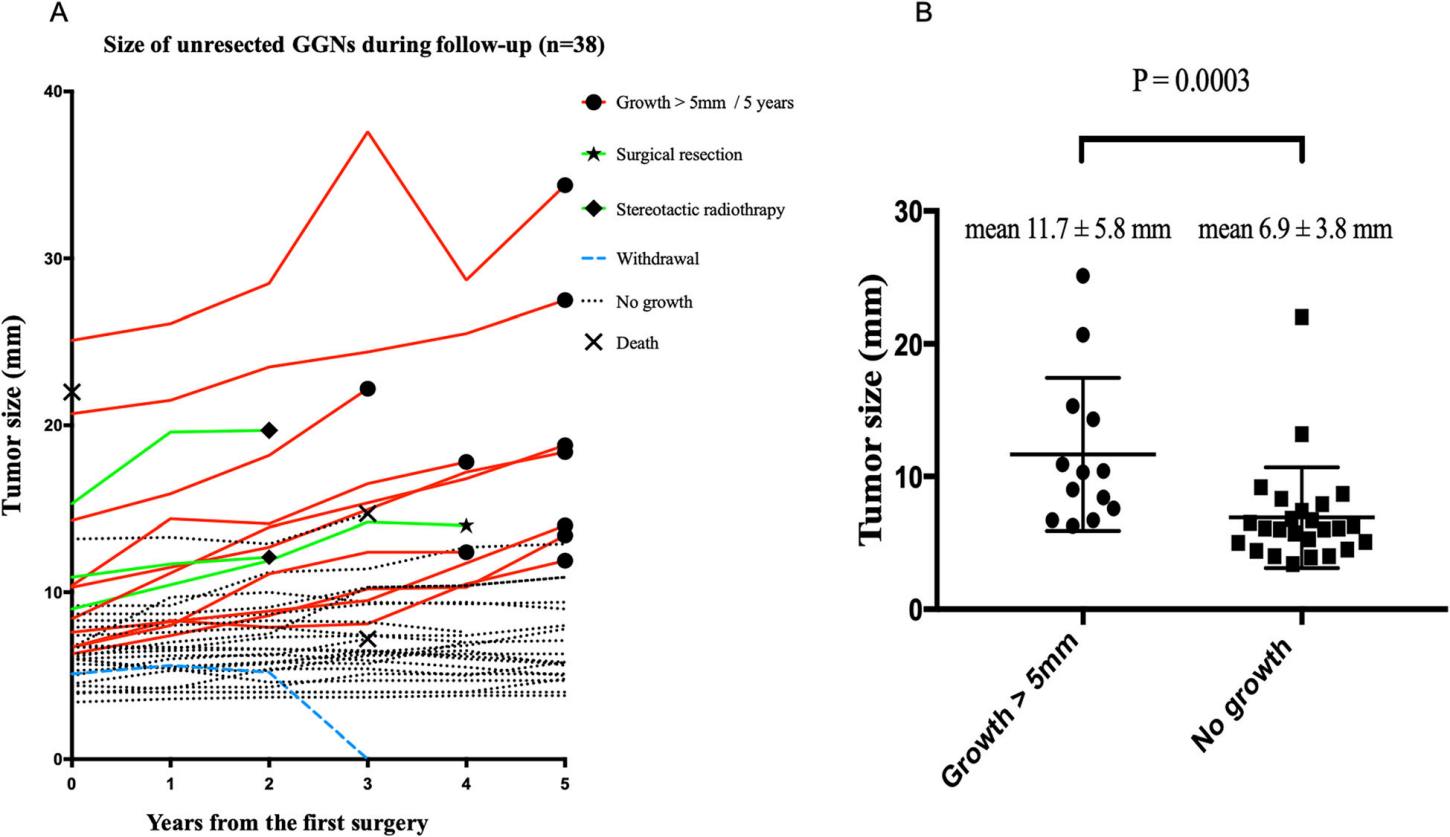
**a** The transition in the size of 38 unresected GGNs on CT. **b** Comparison of the tumor diameter before surgery between lesions with and without growth. The preoperative tumor size of GGNs was significantly larger in comparison to tumors that did not grow in size (mean size, 11.7 ± 5.8 mm vs. 6.9 ± 3.8 mm, *P* = 0.0003). GGNs; ground glass nodules

The transition of the size of the 38 unresected GGNs on CT is shown in Fig. 5a. Twelve GGNs fulfilled the growth condition. Among them, 10 GGNs showed a change in size changed within 3 years. It took four years for all lesions change in size. The tumors of the GGNs that changed in size were significantly larger than no growth tumors (mean size 11.7 ± 5.8 mm vs. 6.9 ± 3.8 mm, *P* = 0.0003) (Fig. 5b).

## Discussion

In this study, we compared multifocal GGNs and single lesions. The number of GGNs had no impact on the OS. As in the case of single lesions, the OS changed according to the pStage. The increase in residual lesions and the appearance of new GGNs were not associated with the OS.

Several studies have reported similar results, showing that prolonged survival was generally achieved by anatomic resection of the DT and wedge resection of the accessible GGNs [11, 14–16]. As for OS and DFS, our results were similar to those of several previous studies. The novelty of this study was that it directly compared the outcomes of patients with multiple GGNs to those of patients with single lesions. The DTs were divided into three categories based on the C/T ratio, and the OS and DFS graphs for multiple and single lesions overlapped. In patients with multiple GGNs, management should be determined based on the DT with the worst prognosis.

Regarding unresected GGNs, previous studies have shown that many lesions remained unchanged, but that a certain proportion increase in size. No relationship was found between the prognosis and progression of the unresected GGN in this study, which is in line with previous reports [15, 16]. In order to reduce wasteful follow-up, we think that is necessary to screen patients and lesions that are likely to increase in size. In this study, we found that the larger unresected GGN tended to increase in size. As far as the lesion was concerned, we found that lesions that were larger in size were more likely to grow. In previous reports, the size of the DT and the proportion of the solid component were also associated with an increased risk of lesion growth [15, 16]. When possible, resecting larger-sized lesions with the DT may be the most efficient approach.

Regarding the follow-up period, GGNs may take 3–4 years to begin to increase in size [17–19]. The same period was considered to be necessary in this study. Patients should be followed up for the same period.

Adenocarcinoma with EGFR mutation is reported to be associated with a higher incidence of GGN in comparison to adenocarcinoma with wild-type EGFR [20]. Many of the recurrent cases in this study had EGFR mutations. Even in early-stage lung cancer, if patients have GGN lesions, the EGFR gene mutation status should be investigated during follow-up. However, it is worth noting that there are many reports of cases in which genetic analyses revealed differences between the DT and synchronous GGNs [10].

The present study was associated with some limitations, including the biases associated with the lack of randomization, as well as the relatively small sample size and limited statistical power. We were unable to analyze the effect of the mutation status. The strength of this study was that it compared the outcomes of patients with multiple GGNs to those of patients with a single lesion. There were no differences between the groups with regard to the methods of treatment and follow-up, and it is was considered to be appropriate as a comparative group.

## Conclusions

The postoperative outcome of lung adenocarcinoma with synchronous GGN was good. 

The outcomes were best predicted by the stage of the DT. After surgery, patients require careful follow-up because unresected GGNs grow in size. At the same time, it is also true that many patients may not require follow-up.

## Data Availability

Not applicable.

## Abbreviations

CT: Computed tomography
GGNs: Ground glass nodules
DT: Dominant tumor
OS: Overall survival
DFS: Disease-free survival
C/T: Consolidation-to-tumor
SRT: Stereotactic radiotherapy
EGFR: Epidermal growth factor receptor
